# Our Closing Reply on Trade Journalism

**Published:** 1889-12

**Authors:** 


### OUR CLOSING REPLY ON TRADE JOURNALISM.

The *Cosmos* has allowed the whole question to go by default, with the exception of the one paragraph in which we tried to explain why the company publisher of the *Cosmos* would not allow its organ to enter into an aggressive fight in the interest of the profession.

No argument has been advanced in answer to our first editorial, in which was discussed the evident dereliction of duty upon the part of that journal towards the profession; neither has any attempt been made to answer our query as to "who made the editor of the *Cosmos* censor of the profession as to what its members shall or shall not discuss in their meetings and journals."

The two editorials written in answer to our strictures have labored hard to make the matter a personal one. The first editorial began with a questionable story, which ended with the following words: "Some of them do, and the rest mind their own business," to which was gratuitously added (italics ours),—

> "We would respectfully refer the editor of the INTERNATIONAL DENTAL JOURNAL to this incident as conveying a lesson which it would do him no harm to consider. . . . It will require something more than the *impudent criticisms* of the editor aforesaid to lessen the confidence of its nearly eight thousand readers. . . . While it is not uncommon for a neophyte to be over-zealous, there can be no excuse for *deliberate* and *officious intermeddling* and misrepresentation. . . . We exceedingly dislike personal controversies with contemporary journals, but when an editor deliberately and repeatedly goes out of his way to criticise the conduct of the *Dental Cosmos* and to assign motives there for, we submit that he violates the rules of polite journalism, and invites the hint *that he had better attend to his own business and let other people's alone.*

To all of which an impersonal and unimpassioned answer was made, with the effect of calling out the following:

> "We took occasion in the *Dental Cosmos* for October to counsel the editor aforesaid that success in journalism was not to be obtained by detraction and misrepresentation of his contemporaries, *and advised him that he would be better engaged in attending to his own business than in meddling in that of his neighbors.* In his November issue he claims that the article which we criticised was written from 'an entirely *impersonal* point of view,' and expresses surprise and regret at the tone of our rejoinder, which he considers *personal, as we meant that he should.*"

The same editorial pronounces ours as "*unadulterated impudence*," and closes with this clause,—

> "Taking leave to add that this commentary is not impersonal."

We are perfectly willing to drop the question on the presentment, and abide the decision of the profession, being fully aware that it "prefers to use its own common-sense" and draw its own inferences.

